# Association between psychological capital and depressive symptoms during COVID-19: The mediating role of perceived social support and the moderating effect of employment pressure

**DOI:** 10.3389/fpubh.2023.1036172

**Published:** 2023-03-09

**Authors:** Yalian Huang, Xin Lin, Jian Yang, Hefei Bai, Ping Tang, Guangzhe Frank Yuan

**Affiliations:** ^1^Sichuan Research Center of Applied Psychology, Chengdu Medical College, The First Affiliated Hospital, Chengdu, China; ^2^School of Psychology, Chengdu Medical College, The First Affiliated Hospital, Chengdu, China; ^3^School of Clinical Medicine, Chengdu Medical College, The First Affiliated Hospital, Chengdu, China; ^4^Department of Health Promotion, Education, and Behavior, Arnold School of Public Health, University of South Carolina, Columbia, SC, United States

**Keywords:** psychological health, depression, employment pressure, medical students, moderated mediation

## Abstract

**Introduction:**

The Coronavirus Disease 2019 (COVID-19) pandemic affects individuals' mental health that can result in fear of getting COVID-19 infection and depression. Prior research has demonstrated that both psychological capital and perceived social support are related to the severity of depression. Yet no study explored the direction of associations between these factors. This undermines the validity of psychological capital as a basis for health interventions.

**Methods:**

This study aimed to explore the association between psychological capital, perceived social support, employment pressure, and depressive symptoms during COVID-19. A cross-sectional design was employed in a sample of 708 Chinese senior medical students who were asked to complete an online questionnaire survey.

**Results:**

Results indicated that psychological capital negatively predicts depressive symptoms (β = −0.55, *p* < 0.001); perceived social support plays a mediating role in the impact of psychological capital on depressive symptoms (indirect = −0.11, *SE* = 0.02, *p* < 0.001, 95%CI [−0.16, −0.07]), and these associations were moderated by employment pressure. Medical students with high employment pressure, the negative impact of psychological capital on depressive symptoms was statistically significant (β = −0.37, *SE* = 0.05, *p* < 0.001, 95% CI [−0.046, −0.27]); when the perceived employment pressure was low, the negative effect of psychological capital on depressive symptoms, although significant, was stronger (β = −0.49, *SE* = 0.04, *p* < 0.001, 95% CI [−0.57, −0.40]).

**Discussion:**

The current study highlights that it is of great significance to address Chinese medical students' employment pressure and improve their mental health during the COVID-19 epidemic.

## Introduction

The employment of college graduates concerns individual careers, family harmony, and the socioeconomic future of society as a whole. Research at home and abroad shows that employment pressure is one of the main stressors of college students (1). According to the 2021 Health Statistical Yearbook of China (2), from 2016 to 2020, there were about 5.96 million new graduates of ordinary higher medical specialties in China, however, the number of health personnel in China increased by about 2.3 million during this period. The increment of medical graduates far exceeds the number of new accommodators in medical and health institutions, and the employment situation of medical students is tight. In 2020, Coronavirus Disease 2019 (COVID-19) spread worldwide, becoming a major public health event with severe consequences on human life and health. In order to stop the spread of COVID-19, the government has taken measures to delay students' return to school and hold online job fairs, which make it difficult for students to find a job and employment pressure is exacerbating (3). Previous study showed that medical students with high levels of concern about the negative impact of COVID-19 on their employment were more likely to report poor sleep quality (4). In the face of global changes brought upon by the pandemic, there is an urgent need to address the psychological wellbeing of medical students with uncertain employment prospects.

### Depression and psychological capital

Depression is the most common health problems, and it is also one of the crucial factors in mental health (5). A cross-sectional study carried out in early 2020 (hence reflecting attitudes in the context of COVID-19) showed that the prevalence of depression in China was 20.1% (6), indicative of the severe impact of the pandemic on depression in the country. In a systematic review of depressive symptoms among medical students during the COVID-19 pandemic, the overall prevalence of depressive symptoms among medical students was reported to be higher than that in other university students (7). Psychological capital refers to a positive psychological state in the process of individual growth and development, including four dimensions of self-efficacy, optimism, resilience, and hope (8, 9).

Psychological capital can predict depression (10). The broaden-and-built theory denotes that psychological resources (e.g., psychological capital) can help individuals form positive emotion regulation strategies against negative emotions (11). Previous study on veterinary medicine interns has also shown that psychological capital plays a protective role in resisting depressive symptoms (12). The depressive symptoms in the early stages of clinical depression are easily overlooked, thus missing the opportunity to prevent progression to clinical depression in time. Psychological capital intervention (PCI) can improve the symptoms of depression (13), which further shows the positive effects of psychological capital on depressive symptoms. Therefore, the current study assumed that psychological capital might be inversely related to depressive symptoms.

### Perceived social support as a mediator

Perceived social support, which is defined as an individual's expectation and evaluation of social support (14). Many studies have shown a positive correlation between perceived social support and psychological capita (15). Empirical studies also found that the psychological capital of volunteers has a positive predictive effect on perceived social support (16). Besides, physicians with higher levels of psychological capital are likely to perceive and take advantage of emotional or material support from leaders, colleagues, and family members (17). In addition, perceived social support was found to be significantly associated with elevated risk for depression (18). Higher levels of perceived social support were associated with lower depression symptoms during the COVID-19 pandemic (19). Taken together, based on these findings, our study aimed to clarify if perceived social support has a mediating effect on the relationship between psychological capital and depressive symptoms of senior medical students during a major epidemic.

### Employment pressure as a moderator

As mentioned above, psychological capital may relieve the depression degree of depression symptoms. However, not all medical students who have a high level of psychological capital relieve equally in their degree of depression symptom, in which employment pressure might play an important role. Employment pressure is considered an individual's cognitive evaluation of the relationship between himself and the environment and the impact of this relationship in the current employment climate. Ego-depletion theory denoted that people's mental resources are limited (20). When faced with employment pressure, individuals will mobilize internal resources to cope with stress; if internal resources are not supplemented in time, the access process is blocked after depletion, which will cause mental health damage. Based on this theory, individuals with high psychological capital have more positive resources to cope with employment pressure, while individuals with low psychological capital are more vulnerable to employment pressure. Research shows that the higher the psychological capital is associated with less perceived employment pressure and better mental health (8, 21). Therefore, this study hypothesized that during the COVID-19 epidemic, the employment pressure of senior medical students would significantly moderate the relationship between psychological capital and depressive symptoms.

### The present study

The present study aimed to investigate the influence of employment pressure, psychological capital comprehensively, and perceived social support on medical students' depressive symptoms and uncover potential mechanisms underlying the links to provide an empirical basis for effective intervention and improvement of medical students' depressive symptoms when facing job hunting. We hypothesized that: (1) psychological capital would have a negative predictive effect on depressive symptoms; (3) perceived social support mediates the relationship between psychological capital and depression symptoms (H2); and (4) employment pressure moderates the relationship between psychological capital and depression symptoms (H3).

## Materials and methods

### Participants and procedure

This study adopted a cross-sectional design, using self-administered questionnaires online. According to the current requirements of COVID-19 prevention and control, we recruited 1,025 senior medical students at the Medical College of Chengdu by convenient random sampling from December 2020 to March 2021. Exclusion criteria for data included: (1) answers to questionnaire questions were incomplete, (3) answers time were under 150s, and (4) answers “no” in the last question of the questionnaire: “Did you answer this questionnaire seriously?.” A total of 312 questionnaires with “No” answers were rejected. Five respondents were removed from the dataset for a completion time under 150's.

We collected 1,025 questionnaires, but only 708 responses were valid, and the effective recovery rate was 69.1%. There were 445 female students (62.9%) and 263 male students (37.1%). The subjects were 19–38 years old (23.14 ± 3.062). 30.8% of the subjects were from cities and 69.2% from rural areas; 68.1% of the subjects are undergraduates, and 31.9% of the subjects are specialties. All study procedures were approved by the Ethics Committee of Chengdu Medical College. Before formally filling in the questionnaire, each subject needs to understand the informed consent form of this study, and then can formally answer the questionnaire after consent. All participants were anonymous and data were kept confidential. Participants who agreed with the informed consent before the online questionnaire survey can undertake the formal task and be compensated after completing the Sichuan Provincial Science and Technology Department Project (20ZDYF2396).

### Measures

#### Depression

Depressive symptoms were measured by the Self-rating Depression Scale (SDS) developed by Zung and William (22). The scale can directly reflect the individual's depressive symptoms, and it is convenient to use. There are 20 items on the subjective feelings of depression. Each item is divided into four grades according to the frequency of symptoms to assess the severity of depressive symptoms. The severity of depressive symptoms is divided into mild depressive symptoms, moderate depressive symptoms, and severe depressive symptoms, with scores of 50–59, 60–69, and 70 or more, respectively. In this study, Cronbach's α coefficient was 0.84, KMO is 0.865, Bartlett's spherical test data *p*-value is at the significance level.

#### Psychological capital

The Positive Psychological Capital Questionnaire (PPQ) (23) has passed the reliability and validity test, and the internal consistency coefficient is 0.90, which has good structural validity. The questionnaire included four dimensions of self-efficacy, resilience, optimism, and hope, with a total of 26 questions. Questions 1–7 represent self-efficacy, questions 8–14 represent resilience, questions 15-20 represent optimism, and questions 21–26 represent hope. A seven-point Likert Scale was adopted for each question. “1” represents strongly disagree, and “7” represents strongly agree. In this study, Cronbach's α coefficient was 0.81, KMO is 0874, Bartlett's spherical test data *p*-value is at the significance level.

#### Perceived social support

Perceived social support was measured *via* the Chinese version of the Perceived Social Support Scale (PSSS) (24). The scale includes three dimensions of family support (items 3, 4, 8, 11), peer support (items 6, 7, 9, 12), and other forms of support (items 1, 2, 5, 10), with a total of 12 items, using 7-point scoring method (1–7 points). The total score of the scale reflects the individual's perception of social support. 12–36 points represent low support level, 37–60 points represent medium support level, 61–84 points represent high support level. The scale includes 12 self-rating items. In this study, Cronbach's α coefficient was 0.78, KMO is 0.833, Bartlett's spherical test data *p*-value is at the significance level.

#### Employment pressure

Employment pressure was measured by the Employment Cognition Evaluation Scale (25). This five-point Likert scale consists of two items, which measure challenge cognitive assessment and threat cognitive assessment.

### Statistical analysis

In this study, IBM SPSS version 17 was utilized to analyze the descriptive statistics. Descriptive analyses were performed using frequency, percentage, and mean ± standard deviation (SD). Means and standard deviations were used to represent continuous variables. One-way analysis of variance (ANOVA) and *t*-tests were used to compare differences between groups. Pearson's correlation coefficient was used to examine the associations between the study variables. Models 4 and 5 in PROCESS version 3.3 developed by Hayes (26) were used to analyses the mediating effect of perceived social support and the moderating effect of employment pressure. We calculated 95% confidence intervals (CIs) based on a 5,000 bootstrap resampling.

## Results

### Common method variance test

The data may suffer from common-method variance since the data collected are electronic self-reports. To mitigate the variance, we completed data collection anonymously and reversed scoring some items (27). After data collection, Harman's univariate factor analysis was used to test whether there were common-method variances. The results showed 17 factors with eigenvalues >1, and the variation explained by the first factor was 17.5%, which was less than the critical standard of 40.0% (28). This shows that there is no serious common-method variance in the data of this study.

### Descriptive statistics and correlation analysis

Descriptive statistics results are shown in Table 1. Pearson correlation analysis showed that depressive symptoms, psychological capital, perceived social support and perceived employment pressure correlated significantly. There is a positive correlation between psychological capital and perceived social support (*r* = 0.57, *p* < 0.01) and a negative correlation between employment pressure (*r* = −0.31, *p* < 0.01) and depressive symptoms (*r* = −0.57, *p* < 0.01). In addition, Table 2 shows a negative correlation between perceived social support and depressive symptom (*r* = −0.48, *p* < 0.01).

**Table 1:** Means, standard deviations, *T*-test, and ANOVA test among study variables.

	**Variable**	***N***	**Psychological capital**	**Perceived social support**	**Employment pressure**	**Depressive symptoms**
Sex	Male	263	111.83 ± 16.944	51.59 ± 10.689	5.96 ± 1.906	48.21 ± 5.277
	Female	445	111.02 ± 18.365	54.26 ± 10.745	5.65 ± 1.348	47.59 ± 5.098
*t*			−0.583	3.20^***^	−2.546^**^	−1.548
Educational background	Undergrade	482	115.4 ± 19.215	55.57 ± 11.631	5.71 ± 1.503	46.55 ± 5.075
	Junior college	226	102.6 ± 9.942	48.36 ± 6.432	5.88 ± 1.744	50.54 ± 4.243
*t*			9.442^***^	0.873^***^	−1.272	−10.279^***^
Permanent residence	City	284	114.39 ± 19.335	55.08 ± 11.94	5.77 ± 1.538	46.64 ± 5.422
	Rural	424	109.26 ± 16.474	52.05 ± 9.781	5.76 ± 1.617	48.61 ± 4.843
*t*			3.788^***^	3.692^***^	0.125	−5.046^***^
Monthly living expenses (¥)	<500	32	107.72 ± 11.229	49.03 ± 8.006	6.03 ± 1.909	50.28 ± 3.401
	501–1,500	395	113.97 ± 18.571	54.92 ± 10.95	5.77 ± 1.544	46.76 ± 5.061
	1,501–3,000	191	107.63 ± 17.076	51.6 ± 10.539	5.76 ± 1.523	48.85 ± 4.909
	>3,000	90	108.79 ± 16.253	51.07 ± 10.289	5.66 ± 1.768	49.4 ± 5.615
*F*			6.826^***^	7.711^***^	0.447	13.98^***^

*** *p* < 0.001;

** *p* < 0.01.

**Table 2:** Means, standard deviations, and correlations among study variables.

**Variable**	**M ± SD**	**1**	**2**	**3**	**4**
1. Psychological capital	111.32 ± 17.84	1			
2. Perceived social support	53.27 ± 10.79	0.57^**^	1		
3. Employment pressure	5.77 ± 1.58	−0.31^**^	−0.21^**^	1	
4. Depressive Symptoms	47.82 ± 5.17	−0.57^**^	−0.48^**^	0.21^**^	1

** *p* < 0.01.

### Mediation analysis

We controlled for the effect of age and analyzed the mediating role of perceived social support in the impact of psychological capital on depressive symptoms. The results showed that psychological capital negatively predicts depressive symptoms (β = −0.55, *p* < 0.001), Hypothesis 1 was thus accepted; psychological capital positively predicts perceived social support (β = 0.54, *p* < 0.001). When psychological capital and perceived social support are used as predictors of depressive symptoms at the same time, psychological capital negatively predicts depressive symptoms (β = −0.44, *p* < 0.001), and perceived social support can also negatively predict depressive symptoms (β = −0.20, *p* < 0.001). The results of mediating analysis showed that perceived social support plays a mediating role in the impact of psychological capital on depressive symptoms (β_indirect_ = −0.11, *SE* = 0.02, *p* < 0.001, 95%CI [−0.16, −0.07]), excluding 0, indicating that perceived social support and was the mediating variables for the psychological capital of medical students to influence depressive symptoms, which accounted for 20.1% of the total effect, as shown in Table 3, Hypothesis 2 was thus accepted.

**Table 3:** Bootstrap results of the mediating effect of perceived social support.

**Effect type**	**Effect value**	**Boot SE**	**Bootstrap 95%CI**		**Proportion of relative effect**
			**Lower limit**	**Upper limit**	
Total effect	−0.55	0.03	−0.61	−0.50	100%
Direct effect	−0.44	0.04	−0.51	−0.37	79.93%
Indirect effect	−0.11	0.02	−0.16	−0.07	20.07%

### Moderated mediation model

**Figure 1:**
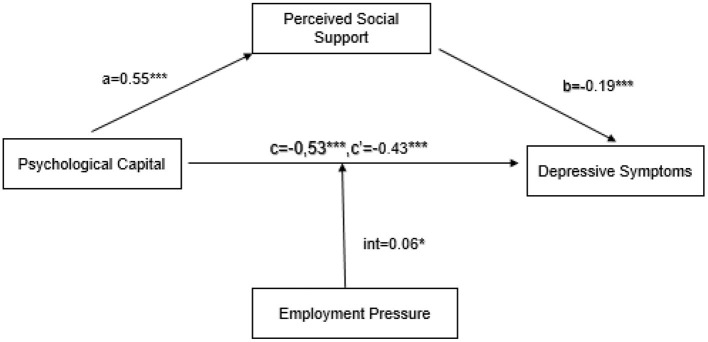
The moderated mediation model. ***p < 0.001, *p < 0.05.

Taking psychological capital as the independent variable, depressive symptoms as the outcome variable, perceived social support as the mediator variable, and perceived employment pressure as the moderating variable, the model was analyzed with model 5 of the PROCESS macro. The overall model is statistically significant (*r*^2^ = 0.37, *F*_(df = 702)_ = 83.42, *p* < 0.001). The direct effect of psychological capital on depressive symptoms is statistically significant (β = −0.43, *p* < 0.001). In the case of perceived high employment pressure (1 SD above the mean), the negative impact of psychological capital on depressive symptoms is statistically significant (β = −0.37, *SE* = 0.05, *p* < 0.001, 95% CI[−0.46, −0.27]); when the perceived employment pressure is low (1 SD below the mean), the negative effect of psychological capital on depressive symptoms, although significant, was stronger (β = −0.49, *SE* = 0.04, *p* < 0.001, 95% CI[−0.57, −0.40]). In addition, the direct (β = −0.19, *p* < 0.001) and indirect effects (β = −0.10, *p* < 0.001, 95% CI [−0.15, −0.06]) of perceived social support on depressive symptoms are both statistically significant, as shown in Figure 1, and Hypothesis 3 was accepted.

## Discussion

### The mediating role of perceived social support

The study showed that psychological capital was positively correlated with perceived social support, and negatively correlated with perceived employment pressure and depressive symptoms, while perceived social support was negatively correlated with depressive symptoms (10); in the relationship between the two, perceived social support acted as a mediator, which showed that perceived social support provides more paths consistent with previous studies (10, 29). In addition, the study revealed that perceived social support partially mediated the effect of depressive symptoms alleviated by psychological capital. In other words, psychological capital can act as a protective factor further combat depression by increasing perceived social support. According to the conservation of resources theory, psychological capital as a psychological resources, could promote youths to pursue wellbeing, even when these youths were under the pressure of life (30), as previous study shows that occupational stress can indirectly affect depressive symptoms through psychological capital (31). The current study indicates that medical students with higher levels of psychological capital are likely to be better able to cope with problems from academic and employment, to perceive and take advantage of emotional or material support from schools, teachers, classmates, and family members, and to against negative emotions. The results of the current study may contribute to a more nuanced understanding of the broaden-and-built theory (11).

Previous studies have proved that quarantine during COVID-19 can result in mental health problems (32, 33), since the time interval of the online questionnaire was from December 2020 to March 2021 when many places in China advocated staying put during Spring Festival (34). Influenced by COVID-19 as a population-wide stressor, all dimensions of individual psychological capital (hope, resilience, optimism, and self-efficacy) were affected to weaken its protective effects on the deterioration of mental health. Therefore, in the context of COVID-19, this study suggested that senior medical students need not only to have higher psychological capital to maintain mental health, but also to enhance their perception of social support, and to actively face their environment. At the same time, this result also provided practical guidance for educators and counselors in the detection and potentially prevented the exacerbation of depression.

However, perceived social support was implemented in the current study as a surrogate measure of actual social support. The correlation between the two was low, and their effect on individual mental health was different (35). From the existing research, the actual social support had no consistent and beneficial effect on the individual's mental health, and may even become a burden to the individual (36). Therefore, what was worth further investigation was how the actual social support impacts mental health in the case of sudden public health emergencies such as COVID-19.

### The moderating effect of perceived employment pressure

The findings showed that the perceived employment pressure of senior medical students had a statistically significant moderating effect on psychological capital and depressive symptoms under COVID-19. Improving psychological capital of individuals can promote their mental health and relieve depression (37). Interestingly, the study found that the influence of psychological capital may not be the same at all levels of depressive symptoms. The alleviating effect of psychological capital on depressive symptoms is contingent on one's level of perceived employment pressure.

Senior medical students under the condition of perceived high employment pressure, psychological capital plays a greater role in. alleviating of depressive symptoms. However, for medical students with perceived low employment pressure, the beneficial effect of psychological capital is enhanced. These findings corroborated with previous research (5). Seligman pointed that positive psychological resources which act as buffers or coping mechanisms could help mitigate potentially stressful situations (38). Therefore, effective and sufficient individual resources can buffer or weaken the negative outcomes caused by employment stress, and reduces the damage to mental health. This result implies that appropriate and moderate perceived employment pressure can improve an individual's mental health.

Existing studies agree that the mental health status of students in clinical medicine is worse than that of non-clinical students (39). Previous study suggests that the components of psychological capital can be learned and strengthened through deliberate interventions (12, 40). Therefore, it is necessary to strengthen career planning guidance for clinical students in medicine, improve the level of psychological capital, and alleviate psychological problems such as depression caused by employment pressure. The observation that higher depressive symptoms are associated with low psychological capital and perceived lower employment pressure is not adequately explored in the current study and should be examined in the future.

## Conclusion

Our findings indicated that psychological capital and perceived social support have a close relationship with depressive symptoms, and perceived social support plays a mediated role between psychological capital and depressive symptoms. Therefore, we recommend that the practitioners of psychological counseling and education be aware of the importance of psychological capital and social support and utilize these resources to mitigate the development and exacerbation of depression. Second, perceived employment pressure moderates the effect of psychological capital on depressive symptoms. Under perceived high employment pressure, psychological capital can predict depressive symptoms, which suggests that psychological capital on mental health cannot be ignored under high employment pressure.

## Data availability statement

The raw data supporting the conclusions of this article will be made available by the authors, without undue reservation.

## Ethics statement

The studies involving human participants were reviewed and approved by Sichuan Research Center of Applied Psychology, Chengdu Medical College. The patients/participants provided their written informed consent to participate in this study.

## Author contributions

PT and GY did study design and data collection. YH and XL analyzed data, drafted, and submitted this manuscript together. PT, GY, JY, and HB revised the manuscript. All authors contributed to manuscript checking and approval the final manuscript.
